# Radiocarbon dating of two old African baobabs from India

**DOI:** 10.1371/journal.pone.0227352

**Published:** 2020-01-16

**Authors:** Adrian Patrut, Arti Garg, Stephan Woodborne, Roxana T. Patrut, Laszlo Rakosy, Ileana Andreea Ratiu, Daniel A. Lowy

**Affiliations:** 1 Babeş-Bolyai University, Faculty of Chemistry and Chemical Engineering, Cluj-Napoca, Romania; 2 Botanical Survey of India, Central Regional Centre, Allahabad, Uttar Pradesh, India; 3 iThemba LABS, Johannesburg, South Africa; 4 Babeş-Bolyai University, Faculty of Biology and Geology, Cluj-Napoca, Romania; 5 Valor Hungariae, Deptartment of Science and Innovation, Budapest, Hungary; Banaras Hindu University, INDIA

## Abstract

The article presents the radiocarbon investigation of the baobab of Jhunsi, Allahabad and the Parijaat tree at Kintoor, two old African baobabs from northern India. Several wood samples extracted from these baobabs were analysed by using AMS radiocarbon dating. The radiocarbon date of the oldest samples were 779 ± 41 BP for the baobab of Jhunsi and 793 ± 37 BP for the baobab of Kintoor. The corresponding calibrated ages are 770 ± 25 and 775 ± 25 calendar years. These values indicate that both trees are around 800 years old and become the oldest dated African baobabs outside Africa.

## Introduction

The African baobab (*Adansonia digitata* L.), belongs to the Bombacoideae subfamily of Malvaceae and is the best-known of the eight or nine species of the *Adansonia* genus [1–4]. The African baobab is endemic to the tropical arid savanna of the African continent between the latitudes 16° N and 26° S. Its current distribution throughout the tropics covers several African islands and different areas outside mainland Africa, where it has been deliberately introduced [1,2,5,6].

An extensive research project was started in 2005 by Patrut et al. in order to clarify several poorly understood aspects on the morphology, development and age of the African baobab. This research relies on a novel methodology, which is not restricted to demised individuals but also allows the investigation and dating of live standing specimens. The original approach described by Patrut et al. is based on AMS (accelerator mass spectrometry) radiocarbon dating of tiny segments extracted from wood samples collected from inner cavities, deep incisions/entrances in the trunk, fractured stems and from the outer part/exterior of large baobabs [7–14].

Baobabs usually start growing as single-stemmed individuals and develop into multi-stemmed trees over time, due to their ability to generate periodically new stems. By this special characteristic, old baobabs typically exhibit very complex and unexpected architectures [10,13]. Thus, our research mainly focused on the study of so-called superlative individuals, i.e., very big and/or old baobabs. Traditional dendrochronological methods cannot be applied to accurately determine the architectures and ages of such superlative trees [10]. The oldest dated African baobabs were found to be over 2000 yr old [10–14]. By these values, the African baobab becomes the longest living angiosperm.

Baobab trees are considered to be a rare sight in India. However, the African baobab is relatively well distributed in states with a tropical or subtropical climate. As a result of surveys, the existence of century-old and heritage specimens has been documented in Andhra Pradesh, Karnataka, Gujarat, Madhya Pradesh, Maharashtra, Rajasthan and Uttar Pradesh. These specimens confirm an antique association of the tree with India.

The introduction of the baobab from Africa to the Indian subcontinent and other regions of the Indian Ocean is a silent and historic affair. The exact route and period of introduction has not been recorded and, practically, little is known about their arrival in India. It is believed that the African baobab was brought to India especially by Arabian sailors who came to establish trade links with regions of the Indian Ocean. Portuguese, Dutch and French invasions may have contributed to the introduction of this species in India. Several studies have also speculated that baobab pods may have floated across from Africa on sea currents, reaching the Indian shores [15–19].

Recent genetic research indicates multiple introduction episodes from various areas of Africa to India, supposedly dating back to prehistoric times [20]. The genetic analysis offered novel and very interesting results. First, it showed that the Indian baobabs are the same species as the African species *Adansonia digitata*. Second, there is less genetic diversity in the Indian baobab populations as compared to the African populations. This confirms the supposition that the baobabs have not been in India long enough for the populations to diversify; thus, their dispersal by ocean currents is less likely than their introduction by humans. The latter discovery indicates multiple introductions of baobabs to India and demostrates they have several biogeographical origins in Africa. Many Indian baobabs have phylogenetic relationship with baobab populations from coastal Kenya and Tanzania, some of them show closer relationship with baobabs from coastal and inland Mozambique and also from West Africa [20,21].

By combining the genetic findings with archaeological and historical accounts of the Indian Ocean trade, Bell and Rangan identified four major periods during which Africans from different regions would have travelled to India. The earliest interactions and transportation of the African baobab to India go back more than 4,000 years ago, when cereals and legumes arrived in India from Sudan, Ethiopia and the Horn of Africa. The second interaction between Africa and India occurred from the 10^th^ to the 17^th^ centuries via the Swahili-Arab trade networks. The third major interaction occured in the 17^th^ and 19^th^ centuries owing to the Portugese, who established their colonial bases in Mozambique and western and southern India. Finally, the fourth interaction associated with the last introduction of the African baobab to India occured over the 18^th^ and 19^th^ centuries, when English and Dutch colonial authorities recruited soldiers from West Africa for regiments in southern India. Thus, one can state that the geographical distribution of baobabs in India is associated with the long history of African diaspora across the Indian Ocean [20,21].

Today India hosts several large and relatively old baobabs. Recently, we investigated and dated by radiocarbon the biggest baobab outside Africa, which is located at Golconda Fort, Hyderabad, India. We found that the oldest part of this very large tree, with a girth of 25.48 m, is around 475 yr old [22].

Here we present the radiocarbon investigation results of two sacred African baobabs from India, namely the baobab of Jhunsi, Allahabad and the Parijaat tree at Kintoor.

## Materials and methods

### 2.1. Ethical statement

The on-site investigation and sampling of the baobabs was authorised by the National Biodiversity Authority of India (NBA/9/2269/18/18-19). The Botanical Survey of India, Central Regional Centre of Allahabad, Uttar Pradesh, provided scientific assistance in this investigation. After each coring, the increment borer was cleaned and disinfected with methyl alcohol. The small coring holes were sealed with Steriseal (Efekto), a special polymer sealing product, for preventing any possible infection of the trees.

The co-author performing the sampling in Fig 6 has given written informed consent (as outlined in PLOS consent form) to publish the photograph.

### 2.2. The two baobabs and their areas

The two trees are located in northern India, in the Uttar Pradesh state. Both have a strange shape and structure, which is not typical for the African baobab.

#### 2.2.1. The baobab of Jhunsi, Allahabad

The historic tree is situated in the village of Jhunsi (or Jhusi), a suburb of the city of Allahabad (officially known as Prayagraj), in the Allahabad district. Radiocarbon dating relates Jhunsi to an ancient archaeological site on the left side of Ganges at Prayag, known under the name Andhernagri and Pratishthanpur. The history of the Jhunsi area begins from 7,100 BCE (i.e., before common era or BC). Its past is documented by archaeological evidences that correspond to five cultural phases, ranging from Chalcolithic to the Early Medieval Period. Epigraphical remains discovered here during Maruya, Sunga, Kushana, Gupta and the Medieval Period testify that Jhunsi reached an advanced degree of civilization at an early date. Pratishthan (Jhunsi) is also described in *Kurma Purana* and *Matsya Purana* as the Prayagamandala [23,24].

The baobab grows on a huge mound of mud on the left bank of the holy river Ganges (Ganga), very close to Triveni Sangam, i.e., the confluence of three sacred rivers: Ganges, Yamuna (Jamuna) and the mythical Saraswati. The sacred tree, which belongs to the Muslim community, is positioned at only 5 m from the “maazar” (tomb) of the Sufi Saint Saiyid Shah Sadrul Haq Taqiuddin (1320–1384), popularly called Baba Shaik Taqi [23,24]. It is said that Shaik Taqi had placed his “datun” (twig used as tooth brush) upside down on the ground, which later developed into this tree. The tree was called “vilaiti imli” (which has not been identified) until 1978, when it was identified as an African baobab by Varmah and Vaid. They also measured its girth between 18 and 20 m and mentioned that the tree is said to be 500 years old [23]. Recently, the massive baobab was investigated again by Singh and Garg, who supposed that its age could be between 750 and 1350 years [24].

**Fig 1:**
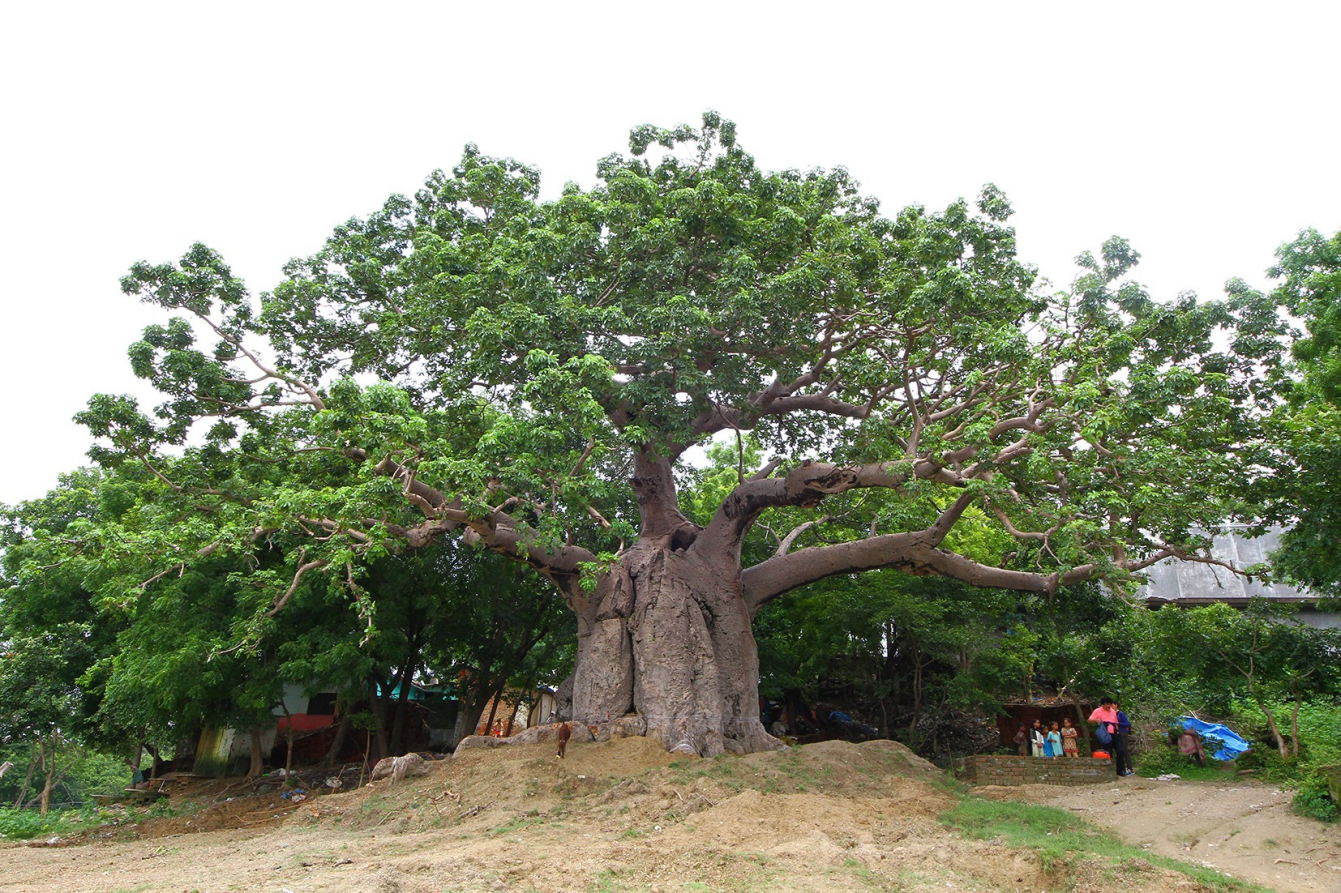
General view of the baobab of Jhunsi, near Allahabad.

The GPS coordinates are 25°25.431’ N, 081°53.981’ E and the altitude is 99 m. The average annual rainfall in the area is 981 mm (Allahabad station) and the mean temperature reaches 25.7°C, with 5–10 frosty days per year (Fig 1).

**Fig 2:**
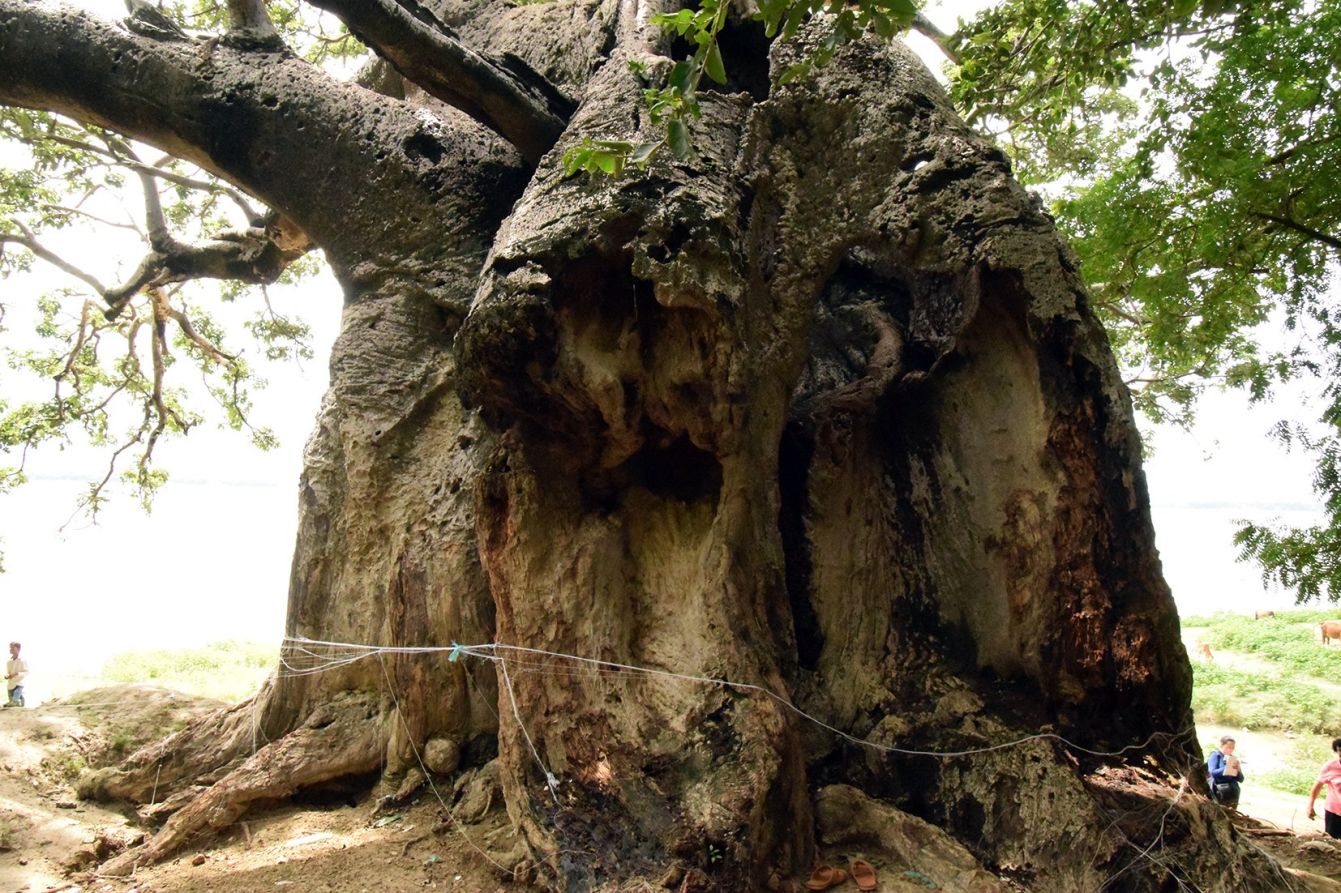
The image shows the two stems of the Jhunsi baobab which were damaged during fire in 2013.

The massive baobab grows on a slope, which tends to widen due to continued errosion by floods of Ganges and water flushing down from adjacent higher areas. The tree was severely damaged during the Kumbh Mela event in 2013, when its eastern side was accidentally set on fire by pilgrims who camped close to the baobab (Fig 2).

The maximum height of the tree is h = 14.0 m. The trunk, with the shape of a haystack, forks at heights of 5.5–7.1 m into 6 primary branches, out of which 3 develop horizontally; at least 4 primary branches are missing. The primary branches have diameters between 0.6–1.2 m. The current circumference of the trunk (at 1.30 m above mean ground level) is cbh = 18.27 m, with two severely damaged and broken stems. We estimate the restored girth, before the fire of 2013, to a value cbh = 21.20 m.

According to our visual inspection, the baobab exhibits a cluster structure and consists of 7 fused stems, out of which 6 are old. The horizontal dimensions of the large hemispherical canopy are 35.9 (NS) x 24.1 (WE) m. The total wood volume of the tree is V = 130 m^3^. The baobab still produces dozens of pods.

The baobab has many hollow parts in the stems and branches. Two relatively large irregular cavities can be observed inside the two damaged stems. It also has 3 big roots, partially exposed due to errosion, which spread along the ground up to 30 m. Some of the bark has been extracted, stripped, sliced and peeled off by locals for medicinal use, especially for treating malaria, cold, flu and diarrhea. These actions exposed the soft tissues to fungal and bacterial attacks.

**Fig 3:**
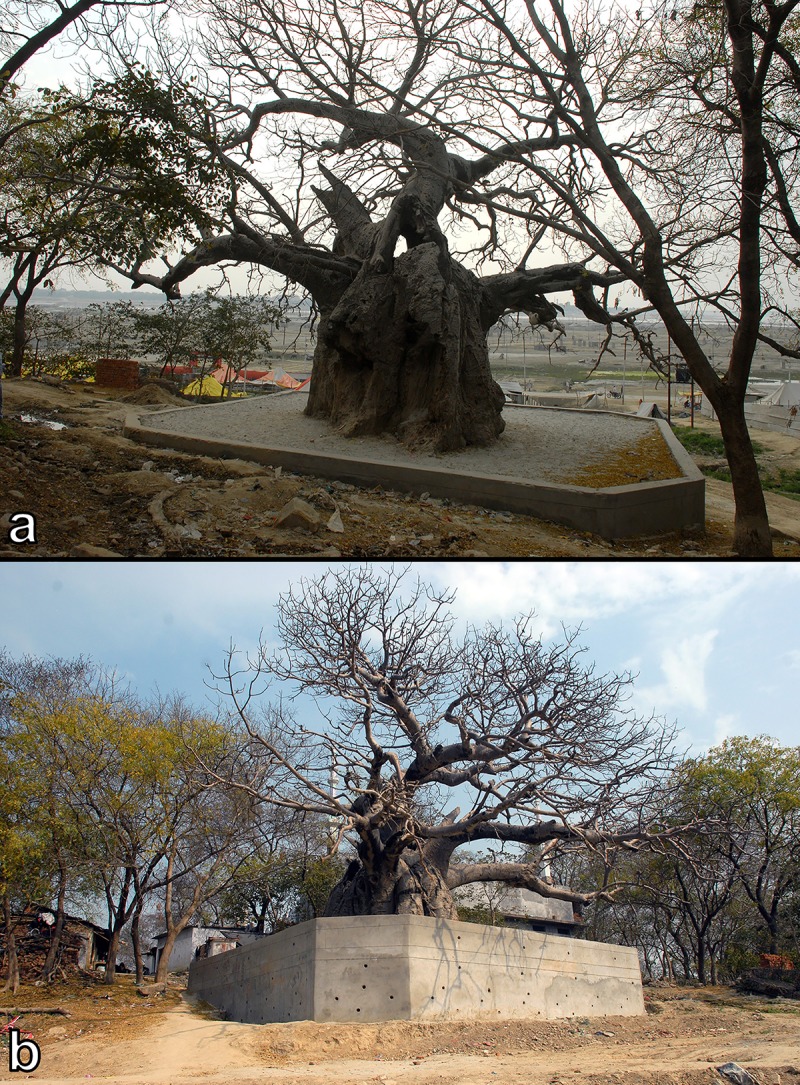
The baobab of Jhunsi with the concrete wall erected around it: seen toward the Ganges (a) and toward the mosque of the village (b).

At the beginning of 2019, the area around the sacred baobab was filled with mud and sand and a concrete boundary wall was erected around the tree for preventing further errosion. The concrete wall has many openings for excess water drain off (Fig 3).

#### 2.2.2. The Parijaat tree at Kintoor

The Parijaat tree at Kintoor, which is worshipped by the Hindu community, has an ancient background. There are many legends around this tree, which might be the most sacred of India. The Parijaat (or Parijat) tree is considered to be a “Kalpavriska” or wish bearing tree, which is only found in heaven. It is claimed that this tree, located near a temple established by Kunti, grows from Kunti’s ashes. Kunti was the mother of the three Pandava brothers, including Arjuna. Arjuna is the main hero of Mahabharata and plays a central role in Bhagavad Gita. According to another saying, Arjuna brought this tree from heaven and his mother Kunti used to crown Lord Shiva with its flowers. The most widespread legend in the area states that Lord Krishna himself brought this tree from heaven, more than 5,000 years ago, for his beloved queen Rukmini.

For a long time, this tree was considered to be the only one of its kind. However, in 1971, Maheshwari identified the Parijaat tree to be an African baobab. He measured its girth to 13 m and estimated its age to be anything from 600 to 5000 years [15].

The Parijaat tree is located at 8 km south of the village of Kintoor (or Kintur) and at 38 km north-east of the Barabanki city, in the Barabanki district. The GPS coordinates are 27°00.206’ N, 081°28.922’ E and the altitude is 106 m. The average annual rainfall in the area is 941 mm (Barabanki station) and the mean temperature reaches 25.6°C, with around 5 frosty days per year.

**Fig 4:**
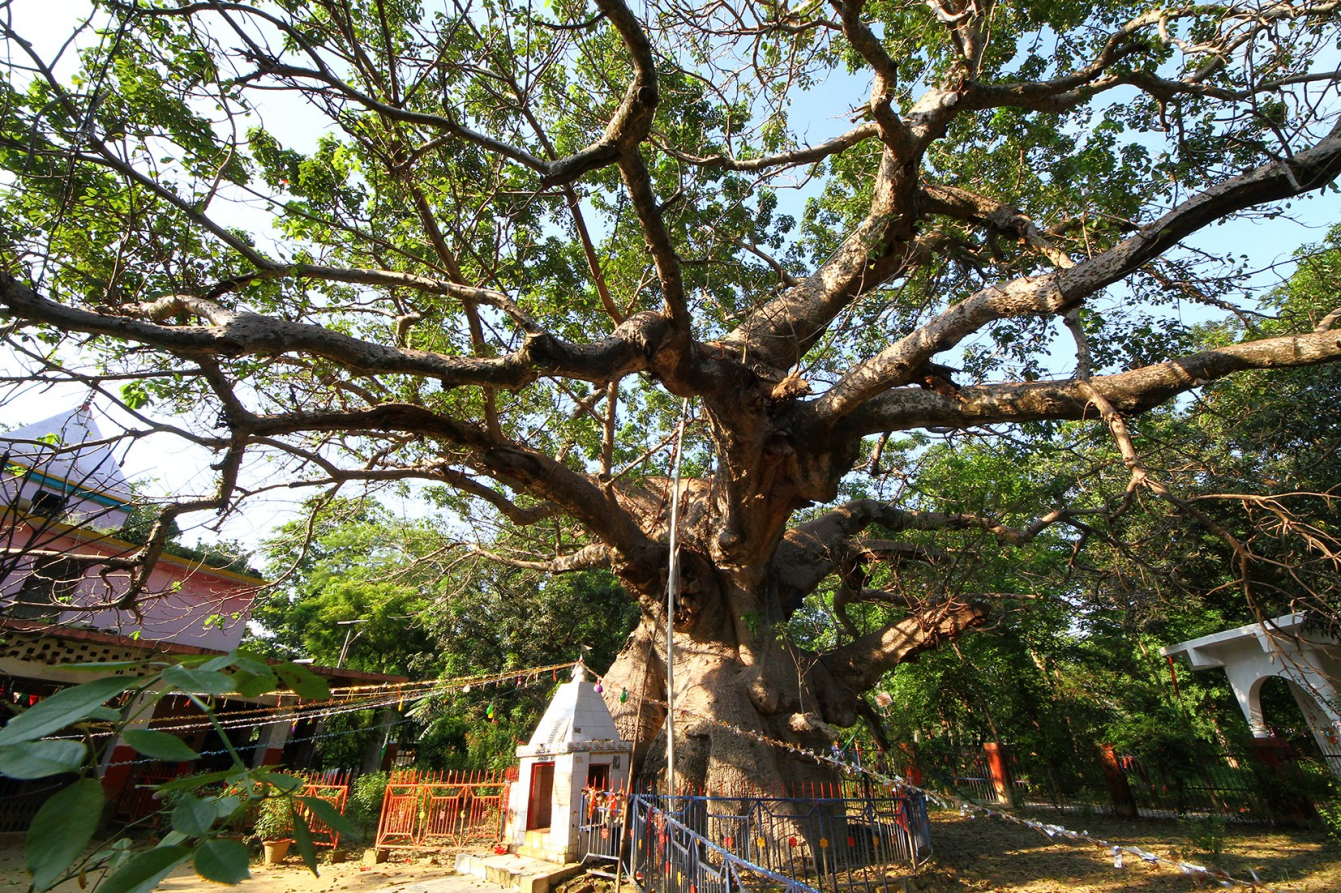
General view of the Parijaat tree at Kintoor.

The tree is situated in a closed complex, which is a place of much religious significance for the locals. The baobab is surrounded by two metal fences. There is a small temple dedicated to Krishna at its base. According to orders of the district magistrate, the temple was fenced off and is not accessible any more (Fig 4).

**Fig 5:**
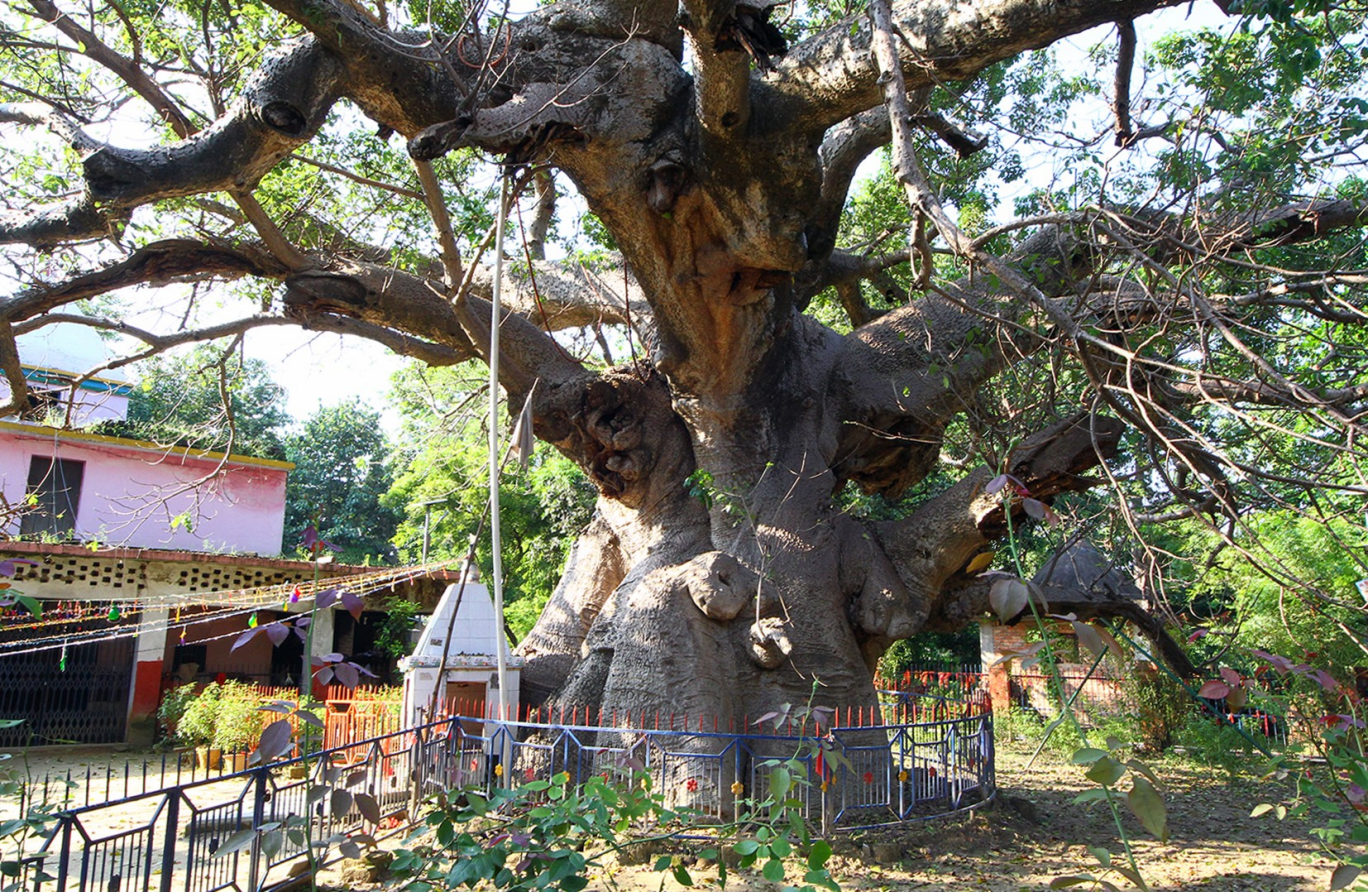
A view of the baobab of Kintoor, highlighting its huge branches.

The maximum height of this tree is h = 13.7 m. The short trunk is of conical shape. It forks at heights of 4.0–5.0 m into 7 very large primary branches, some of them broken, with diameters between 1.2–2.0 m. At least 3 primary branches are missing. The branch sizes are exaggerated in comparison with the relatively modest trunk size (Fig 5). The circumference of the trunk is cbh = 13.09 m, with a partially broken stem. We estimate the restored girth, before this stem split, to cbh = 14.10 m.

The Parijaat tree also exhibits a cluster structure and consists of 4 or 5 perfectly fused stems. The horizontal dimensions of the very impressive canopy are 33.4 (NS) x 31.6 (WE) m. The overall wood volume is of 95 m^3^, out of which 50 m^3^ belongs to the trunk and 45 m^3^ to the canopy. According to locals, over the past century at least, the tree did not produce pods.

### 2.3. Sample collection

**Fig 6:**
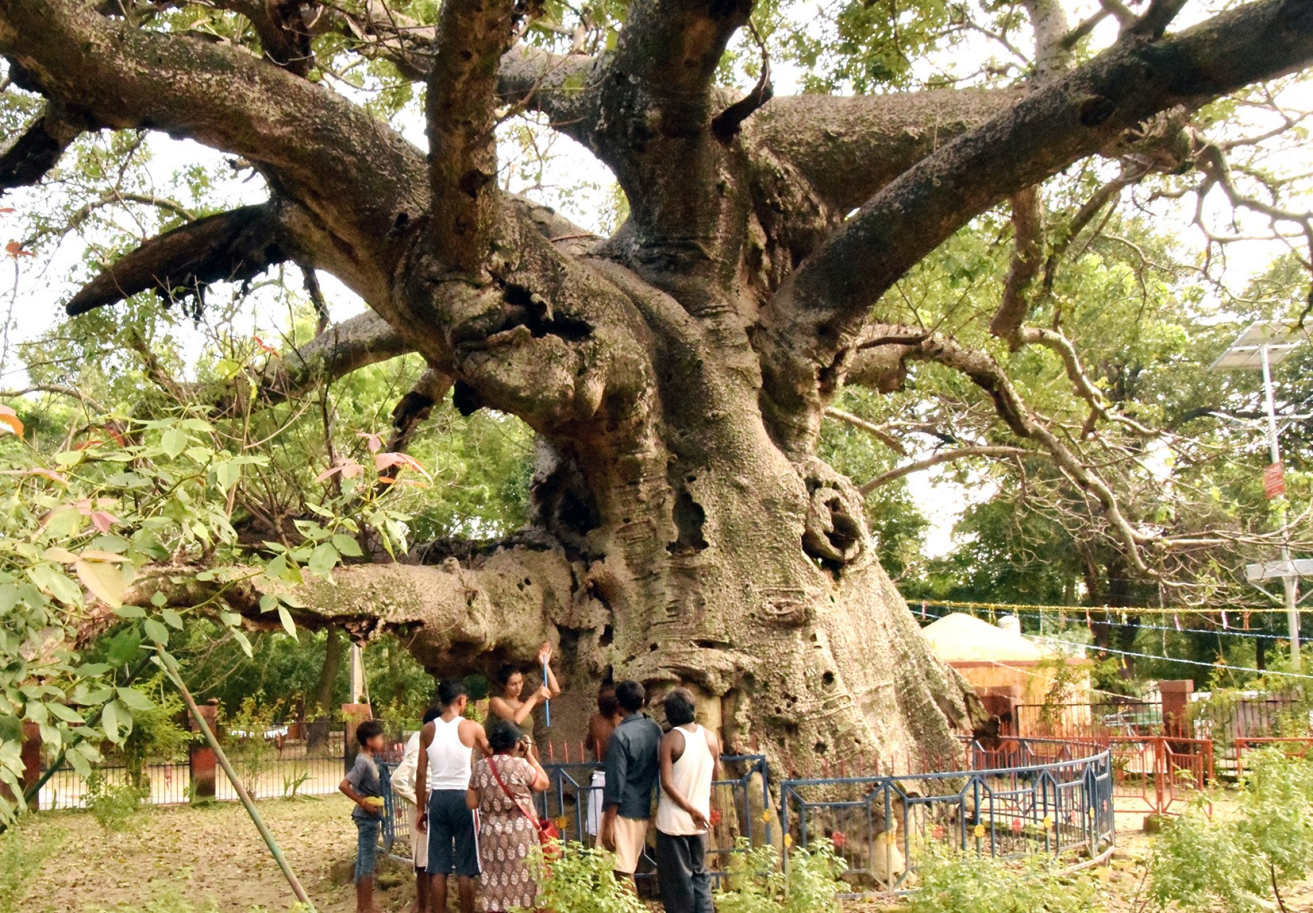
Collecting sample PK-1 from the Parijaat tree.

As agreed with the local religious leaders, one sample was collected with the increment borer (Haglöf; 0.80 m long, 0.010 m inner diameter) from one presumptive old stem of each baobab. These two samples were labelled JA-1 and PK-1 (Fig 6). Several tiny segments, each 10^−3^ m long (named a, b, c), were extracted from predetermined positions/distances along every sample. Additionally, we collected with a sharp instrument three tiny samples from one severely damaged stem of each baobab. These additional samples were labelled JA-2, JA-3, JA-4, PK-2, PK-3 and PK-4.

### 2.4. Sample preparation

The α-cellulose pretreatment method was used for removing soluble and mobile organic components [25]. The resulting samples were combusted to CO_2,_ which was next reduced to graphite on iron catalyst [26,27]. The resulting graphite samples were analysed by AMS.

### 2.5. AMS measurements

The AMS radiocarbon measurements were done at the AMS Facility of the iThemba LABS, Johannesburg, Gauteng, South Africa, using the 6 MV Tandem AMS system [28]. The obtained fraction modern values were finally converted to a radiocarbon date. The radiocarbon dates and errors were rounded to the nearest year.

### 2.6. Calibration

Radiocarbon dates were calibrated and converted into calendar ages with the OxCal v4.3 for Windows [29], by using the IntCal13 atmospheric data set [30].

### 2.7. Water content

The water content of one stem of each baobab was determined by dehydration of wood segments (depth 0.20–0.30 m) for 72 h, at 120°C, under ambient atmosphere.

## Results and discussion

### 3.1. Radiocarbon dates and calibrated ages

Radiocarbon dates, expressed in radiocarbon years BP (before present, i.e., before the reference year 1950) and calibrated ages (expressed in calendar years CE, i.e., common era) of 11 sample segments are listed in Table 1. The 1σ probability distribution (68.2%) was typically selected to derive calibrated age ranges. For three sample segments (JA-4, PK-1b, PK-2), the 1σ distribution is consistent with one range of calendar years. For two segments (PK-1a, PK-4), the 1σ distribution corresponds to several ranges. In these cases, the confidence interval of one range is considerably greater than that of the others; therefore, it was selected as the cal CE range of the sample for the purpose of this discussion.

**Table 1:** Radiocarbon dating results and calibrated ages of samples collected from the baobab of Jhunsi, Allahabad (JA) and from the Parijaat tree at Kintoor (PK).

Tree	Sample/ segment code	Height^1^ (m)	Depth^2^ (m)	Radiocarbon date [error] (^14^C yr BP)	Cal CE range 1σ or 2σ [confidence interval]	Sample/ segment age [error] (cal yr)	Accession #
Baobab Jhunsi Allahabad	JA-1a	1.45	0.12	90 [± 30]	1684–1732 [26.3%] **1807–1928 [69.1%]**	150 [± 60]	IT-C-1673
	JA-1b	1.45	0.18	143 [± 42]	1666–1784 [43.4%] **1796–1894 [34.9%]** 1905- . . .[17.1%]	175 [± 50]	IT-C-1678
	JA-1c	1.45	0.31	188 [± 30]	1649–1694 [21.3%] **1726–1813 [53.1%]** 1918- . . . [21.1%]	250 [± 45]	IT-C-1711
	JA-2	1.96	0.78	-	**-**	>Modern	IT-C-1774
	JA-3	1.94	0.82	-	**-**	>Modern	IT-C-1844
	JA-4	1.98	0.85	779 [± 41]	**1222–1272 [68.2%]**	770 [± 25]	IT-C-1840
Baobab Parijaat Kintoor	PK-1a	1.45	0.15	155 [± 33]	1669–1694 [13.4%] **1727–1780 [31.9%]** 1798–1812 [8.2%] 1918–1945 [14.8%]	265 [± 25]	IT-C-1689
	PK-1b	1.45	0.40	507 [± 29]	**1411–1436 [68.2%]**	595 [± 10]	IT-C-1714
	PK-2	1.10	0.80	793 [± 37]	**1219–1266 [68.2%]**	775 [± 25]	IT-C-1845
	PK-3	1.12	0.75	-	**-**	>Modern	IT-C-1691
	PK-4	1.13	0.73	693 [± 36]	**1272–1300 [52.7%]** 1368–1381 [15.5%]	735 [± 15]	IT-C-1837

^1^Height above ground level.

^2^Depth in the wood from the exterior.

For three sample segments with lower positive radiocarbon dates (JA-1a, JA-1b, JA-1c), the 1σ distribution corresponds to several ranges which have all very low probabilities. In these cases, we used for calibration the higher 2σ probability distribution (95.4%), which corresponds to two or three age ranges. We used the same approach for selecting the cal CE range of each sample segment, with one exception. This exception is the segment JA-1b, for which we selected the range with the second highest probability, which agrees better with the age sequence along the sample JA-1.

In all cases, the selected age range is marked in bold in Table 1.

For three sample segments (JA-2, JA-3, PK-3), ages fall after 1950 CE (0 BP), namely the 14C activity, expressed by the ratio 14C/12C, shows higher values than the standard activity registered in the reference year 1950. These results correspond to negative radiocarbon dates and are named greater than Modern (>Modern). Such cases indicate a very young age of the dated wood, which was formed after 1950 CE.

### 3.2. Sample ages and errors

Sample ages represent the difference between the year 2019 CE and the mean value of the selected age range (marked in bold). The sample ages and the corresponding errors, which are expressed in calendar years, were rounded to the nearest 5 years.

We used this approach for selecting calibrated age ranges and single values for sample ages in all our previous articles on AMS radiocarbon dating of large and old angiosperm trees, especially of baobabs [7–11,13,14,22].

### 3.3. Dating results of samples

The two samples collected with the increment borer were relatively short, i.e., 0.31 m for JA-1 and 0.40 m for PK-1, even if the penetration of the borer was almost complete. This reveals that both trees have large hollow parts in the corresponding stems. The radiocarbon date of the deepest segment was 188 ± 30 BP for JA-1c and 507 ± 29 BP for PK-1b. These values correspond to calibrated ages of 250 ± 45 and 595 ± 10 yr.

For the baobab of Jhunsi, Allahabad, the oldest dated sample JA-4 was collected close to the pith of a stem severely damaged by the fire of 2013. The radiocarbon date of 779 ± 41 BP corresponds to a calibrated age of 770 ± 25 yr. In the case of the Parijaat tree at Kintoor, the oldest dated samples PK-2 and PK-4 were also collected close to the pith of a stem, which broke off several decades ago. Their radiocarbon dates of 793 ± 37 BP and 693 ± 36 BP correspond to calibrated ages of 775 ± 25 and 735 ± 15 cal yr.

The very young ages of three samples (JA-2, JA-3, PK-3), with negative radiocarbon dates, collected from areas close to those of the oldest samples, can be explained by the presence of young regrowth wood for healing produced just after the major damage suffered by the respective stems [8].

### 3.4. Trees/stems ages

The radiocarbon dates and calibrated ages of the oldest dated samples (JA-4, PK-2, PK-4) suggest ages close to 800 yr for the stems from which they originate. Because both baobabs have a cluster structure and consist each of several fused stems with close ages, we consider that the age of the baobab of Jhunsi Allahabad, as well the age of the Parijaat tree at Kintoor, is between 750–850 yr, namely 800 ± 50 yr, which is considerably older than we have expected. One can state that both baobabs started growing around the year 1200 CE.

By these values, both trees become not only the oldest African baobabs from India, but also the oldest baobabs outside Africa with accurate dating results. The former record holder was the Big Biobab of Mannar Town (Sri Lanka), which is around 750 yr old [31].

### 3.5. Water content of stems

The baobab wood has usually a high water content, up to 79%. Big baobabs stay erect/upright mainly due to the weight of stems. When this weight drops to a critical level due to water loss, the stability of the baobab is affected [32,14].

We measured the water content of the stems, which were sampled with the increment borer. We found extremely low values, more precisely 45.2% for the baobab of Jhunsi, Allahabad and only 39.7% for the Parijaat tree at Kintoor. This low values indicate that both baobabs are close to the end of their life cycle and may topple in a near future.

## Conclusion

The research reports the results of the AMS radiocarbon investigation of two sacred African baobabs from the Uttar Pradesh state, in northern India. The two investigated trees are the baobab of Jhunsi, Allahabad and the Parijaat tree at Kintoor. Both baobabs have a cluster structure and consist of several fused stems. Several wood samples were collected from the exterior of stems, as well as from deep areas of severely damaged stems. The oldest samples have radiocarbon dates of 779 ± 41 BP for the baobab of Jhunsi and 793 ± 37 BP for that of Kintoor. These dates correspond to calibrated ages of 770 ± 25 and 775 ± 25 yr. According to these values, the age of both baobabs is close to 800 yr. Thus, the baobab of Jhunsi, Allahabad and the Parijaat tree at Kintoor become the oldest dated African baobabs outside Africa.

The general state of deterioration and the low water content of stems indicate that the two sacred baobabs are in decline, close to the end of their life cycle.
